# Expression of Concern: Water hyacinth: a possible alternative rate retarding natural polymer used in sustained release tablet design

**DOI:** 10.3389/fphar.2016.00062

**Published:** 2016-04-08

**Authors:** Frontiers in Pharmacology Editorial Office

With this notice, we alert readers that Frontiers has received two messages questioning the soundness of the experimental results of the publication “Water hyacinth: a possible alternative rate retarding natural polymer used in sustained release tablet design” published on 11 June 2014 in Frontiers in Pharmacology. Our Chief Editors, Prof. Theophile Godfraind and Dr. Dominique Dubois, will direct an investigation in full accordance with our procedures and with the collaboration of the authors. The situation will be updated as soon as the investigation is complete.

